# Neanderthals *versus* Modern Humans: Evidence for Resource Competition from Isotopic Modelling

**DOI:** 10.4061/2011/689315

**Published:** 2011-09-15

**Authors:** Virginie Fabre, Silvana Condemi, Anna Degioanni, Estelle Herrscher

**Affiliations:** ^1^Laboratoire d'Anthropologie Bioculturelle, UMR 6578 CNRS/Université de la Méditerranée/EFS, Faculté de Médecine-Secteur Nord, Université de la Méditerranée, CS80011 Bd Pierre Dramard, 13344 Marseille Cedex 15, France; ^2^Laboratoire Méditerranéen de Préhistoire Europe Afrique (LAMPEA)-UMR 6636-MMSH 5, rue du Château de l'Horloge-BP. 647, 13094 Aix-en-Provence Cedex 2, France

## Abstract

During later MOIS3, in Europe two populations were present, autochthonous Neanderthals and modern humans. Ecological competition between these two populations has often been evoked but never demonstrated. Our aim is to establish whether resource competition occurred. In this paper, in order to examine the possibility of ecological competition between these two populations, 599 isotopic data were subjected to rigorous statistical treatment and analysis through mixing models. The aim of this paper was to compare dietary strategies of Neanderthals and modern humans over time. Our conclusions suggest that Neanderthals and modern humans shared dietary habits in the particular environmental context of MOIS3 characterised in Europe by climatic deterioration. In this environmental context, the resource competition between Neanderthals and modern humans may have accelerated the disappearance of the Neanderthal population.

## 1. Introduction

The Neanderthals are a well-known middle Pleistocene population, which was autochthonous in Europe during MOIS 6, 5, and 4. The European Neanderthals are associated with Mousterian assemblages. 

During the later part of MOIS3, in the late Pleistocene, Europe was also populated by modern humans. The presence in Europe of modern humans is inferred, according to some authors, in the oldest Eastern European sites by association with Protoaurignacian or Aurignacian assemblages [1] and, for later periods of MOIS3, by association also with fossil remains [2]. The dates available for the Protoaurignacian, Aurignacian, and late Mousterian sites show that, after the arrival of modern humans, there was a period of coexistence between these two populations in Europe for at least 15,000 years [3]. This period is marked by an increase of Aurignacian sites throughout Europe, the appearance of so-called “transitional assemblage” sites (Chatelperronian, Uluzzian, Szeletian, Lincombian, Ranisian, Jerzmanowician), and the decline of Mousterian sites. After 25,000 years BP, the Mousterian sites and the Neanderthal population completely disappeared in Europe, and only modern humans survived on this continent.

Despite numerous investigations, the debate concerning whether Neanderthals became extinct because of climate change or competition with Modern humans is still unresolved. Some researchers argue that competition alone cannot be the cause of Neanderthal extinction [4–7]. By contrast, other authors support the existence of competitive exclusion for the same niche and argue that competition played a major role in the demise of the Neanderthal population. Some analyses, which are based on mathematical modeling, lack plausibility because they are too theoretical [8]; others, which are based on more integrative simulations [9] or which take into account archeological and ethnologic examples [10], are more convincing.

The modelling approach is used to understand complex systems by working on a simplified model of these systems. Thus, this process involves the choice of certain parameters and variables which, if they are simplified, are nonetheless controlled in such a way that they are capable of representing the system as a whole. Therefore, the model used in this paper does not attempt to determine the kind of food that Neanderthals and modern humans consumed but to highlight the potential differences in dietary habits characteristic of these two populations.

The aim of this paper is to test the hypothesis that resource competition, analysed through isotopic modelling, was strong between Neanderthals and modern humans. It assumes that if a model shows similar dietary patterns for Neanderthals and modern humans, then these two populations would be in competition for resources. By contrast, if the models show differences in dietary patterns, this would signify that resource competition would be less intense. 

 For some years, isotopic biochemistry allowed us to improve our knowledge about past human diet using carbon and nitrogen isotopic ratios [11–15]. Since 1990's these methods have been increasingly used to study paleontological populations such as Neanderthals or early modern humans, in order to understand their relationship with the local environment (e.g., [11, 16–23]). Therefore, the literature contains a substantial number of isotopic data, mainly on carbon and nitrogen isotopic values measured on bones and dental collagen. The previous studies suggested that prehistoric peoples had a carnivorous diet similar to that of contemporaneous predators, such as cave lions or cave hyenas [19, 24]. These isotopic studies are consistent with zooarchaeological investigations which showed that Neanderthals and modern humans were big game hunters (hunting mainly big ungulates) [25–30].

The isotopic modelling used in this paper presents a new method of investigation that intends to contribute to the debate on resource competition between Neanderthals and modern humans which has often been assumed but never really demonstrated.

## 2. Material and Methods

### 2.1. Compilation of the Database

**Figure 1:**
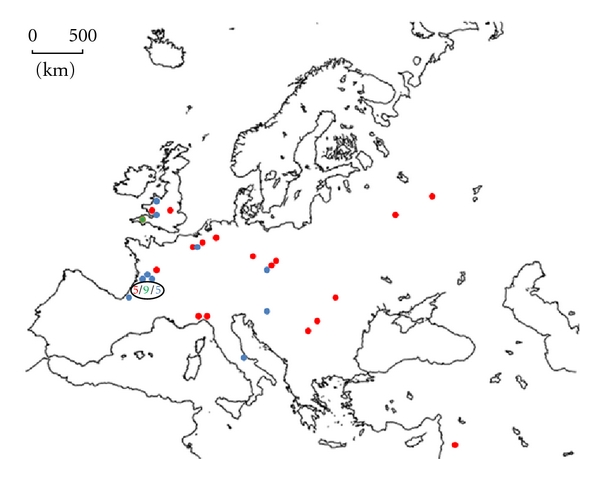
The 51 major archaeological sites providing isotopic data, in red, archaeological sites with only human data, in blue with human and faunal data and in green with only faunal data.

Isotopic data from 51 major archaeological sites in Europe (Figure 1) were compiled from 42 publications. Of these archaeological sites, 14 were attributed to Neanderthal and 37 to modern human settlements. In total, isotopic data from 945 specimens (faunal and human) was assembled from the literature. This paper focuses on the transition between MOIS3 and MOIS2; as such, isotope data from species unavailable during these time periods was eliminated from the data set. Furthermore, the models employed in this paper rely exclusively on three faunal types (reindeer, horse, and bovid) because these were the only remains present at all sites. As a result, only isotope data from 599 specimens were included in this analysis (Table 1 and Tables SI1, SI2, SI3).

As Drucker has shown, local environmental context can influence isotopic signatures of plants and consequently those of consumers [31]. As a result, the first step of our analysis was to verify isotopic modifications for each faunal type through time and space [32]. Thus, in addition to chronology (e.g., late MOIS3 versus MOIS2), data relating to geography and environment were also considered (Table 2). Environmental groupings were in agreement with Allen and Huntley in 2002 [62]. Radiocarbon dates were updated by Jöris and Street 2008 [3].

In order to study the transition between late MOIS3 and MOIS2, we created models by grouping faunal types and humans species into three chronological groups: (1) MOIS3 Neanderthals, (2) MOIS3 modern humans, (3) MOIS2 modern humans. In this paper we used the term “MOIS3” in order to nominate the coevolution period of Neanderthals and modern humans in Europe; thus “MOIS3” here represents the later period of MOIS3. These groups were analysed in three different ways: (1) an absence of cluster (global), (2) a geographical cluster, (3) an environmental cluster. Due to limitations related to the faunal isotopic data available for each cluster and the fact that models have to be run with the same characteristics for diachronic comparison, models were limited to: (1) the whole dataset, (2) the data of south-western area, (3) the data relating to cold environments of tundra-steppe and open boreal woodland (Table 3).

### 2.2. Isotopic Values

Patterns in human and animal food consumption are reconstructed using carbon and nitrogen isotope ratios in bone collagens. Since collagen is protein, the stable isotope ratio of this tissue provides information on the protein component of the diet over approximately the last 10 years of an individual's life [63, 64]. Because plants and animals differ in their carbon and nitrogen isotope ratios it is possible to use their ratio to infer past dietary patterns. Carbon isotope ratios are typically used to differentiate between the consumption of C3 versus C4 plants or marine fish versus fresh water fish [11, 14, 63, 65, 66]. In contrast, nitrogen isotope ratios are indicative of trophic level (i.e., an individual position in the food web) [65, 67]. Stable isotope ratios reflect the type of primary protein sources and are successively enriched in the heavy isotope (^13^C, ^15^N) with each step up the food web [65, 68]. Thus, the relative isotopic variability between different organisms of a terrestrial and aquatic trophic web is distributed in a predictive way from plants at the baseline of the food chain through the subsequent levels as herbivorous and carnivorous organisms. For example, the *δ* ^13^C and *δ* ^15^N values of collagen from herbivores are approximately 5% and 3–5% higher, respectively, than plants [20, 63]. In a similar way, the *δ* ^13^C and *δ* ^15^N values of collagen from carnivores are approximately 0.8–1.3% and 3–5% higher, respectively, than herbivores [19]. According to isotopic data available for the Palaeolithic terrestrial environments, *δ* ^13^C values of plants range from −35 to −20% with a distinction between open and closed environments, and *δ* ^15^N values of plants ranged from 0 to 6%. The *δ* ^13^C and *δ* ^15^N values of collagen from herbivores range from −30 to −18% and 3 to 8%, respectively. The *δ* ^13^C and *δ* ^15^N values of collagen from carnivores range from −24 to −16% and 7 and 13%, respectively. The *δ* ^13^C and *δ* ^15^N values of collagen from freshwater fish range from −23 and −19% and 9 and 15%, respectively.

### 2.3. Modelling Process

Following the predictive fractionation of isotope ratios through the food chain, Phillips and colleagues proposed different isotopic mixing models to quantify the relative contributions of the different dietary sources to an individual [69–71]. IsoError (2001) and IsoConc (2002) based on the isotopic mixing models estimate the proportions for two food sources using a single isotopic element or three sources using two isotopic elements. IsoError mixing model considers the isotopic signature standard deviations in the source and mixture populations and restitutes food proportions with confidence intervals for source proportion estimates. In contrast, the IsoConc mixing model is a concentration-weighted linear mixing model which considers for each element the contribution of source as proportional to the weight of the elemental concentration in that source. The IsoSource mixing model (2005) can estimate more than 3 sources using 2 stable isotopic elements when food sources are isotopically very different. The different isotopic mixing models have previously been applied in past foodwebs to assess the relative proportions of different sources to a single or population human mixtures [33, 72–78].

Based on isotopic mixing model proposed by Phillips et al., the final aim of the research was not to determine the real contributions of specific food sources to a mixture but to compare the human dietary patterns over time and space within distinct chronological groups. Simulations were run with IsoSource mixing models (version 1.3.1) [69, 79]. The use of mixing models to study European Palaeolithic populations could be limited because the mixing models were designed from datasets composed of living North American animals. However, regardless of chronological period and geographical context, the same principle of isotopic fractionation along the trophic chain is generally applicable to all isotopic studies. Furthermore, the mixing models were applied to compare different cohorts according to different parameters and not to precisely reconstruct the proportions of different resources consumed by past human populations. As such, whatever limitations exist will be similar in each group and should not affect the comparisons. 

The use of models necessitates a variety of assumptions. For example, in this study we assume that all the hominids considered consumed the same kinds of resources. Following the recommendation of Phillips et al. [69], only three food sources were considered: fish, meat, and plant resources because they are (i) largely distant from the mixture and (ii) sufficient and reliable to consider the main food items consumed in typical omnivorous diet. Due to the lack of data, plants are not often considered in previous isotopic studies whereas they are necessary for human survival [80]. This resource was included in our simulations permitting to consider one of food items consumed by omnivorous. Due to the isotopic fractionation from diet to consumer (bone collagen), in following simulations, isotopic values of hominid diet, mentioned as “mixing diet” or “mixture”, were considered to be lower than isotopic values of hominid collagens of 0.8–1.3% for carbon and 3–5% for nitrogen [19, 20, 65, 68]. For each cluster datasets, and according to the appropriate fractionation factor requires for running models, isotopic values of plants were estimated from available herbivorous isotopic values (0.8–1.3% for carbon and 3–5% for nitrogen) (see Supplementary Material SI3 available online at doi: 10.4061/2011/689315). Isotopic values of meat and fish resources of hominid diets were derived from the literature and summarized in the database (Table SI1).

**Figure 2:**
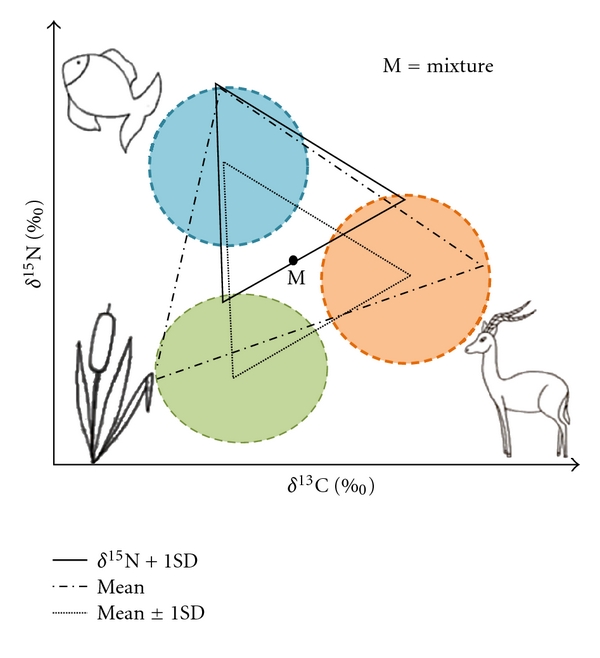
Geometric constructions used for running simulations (the example shows the whole dataset; sources are represented by mean ± 1SD; the values used for running simulations are described in Table SI3).

For each cluster (global, geographical, and environmental), 3 sets of simulations were performed (one for each chronological groups: MOIS3 Neanderthals, MOIS3 modern humans, and MOIS2 modern humans). To get the best inputs for running IsoSource and to have comparable patterns for each chronological group, a similar geometric construction was realised within the estimated carbon and nitrogen variability of each food source (mean ± SD) (Figure 2). The isotopic variability for herbivorous source was calculated by averaging the mean of each faunal type isotopic signature to avoid any bias due to sample size. Moreover, concerning the whole dataset and the cold environments dataset, the geometric construction usable for modelling had to be based on the largest isotopic values of sources around the mixture. Concerning the South-western dataset, two sets of simulations have been performed; the first one involves a geometric construction based on the largest isotopic values of sources around the mixture (mean ± 1SD), and the second one involves a geometric construction based on average isotopic values of sources (mean).

### 2.4. Statistical Analysis

To assess the validity of the different clusters proposed in Section 1, a set of Kruskall-Walis statistics was applied to the database (Statistica software). Kruskall-Wallis statistics are nonparametric tests generally used to compare the distribution of two independent sets of values. Here the aim is to test (i) if chronological groups are reliable for investigating the homogeneity of stable isotopic signatures of each faunal group across the time and (ii) if all archaeological sites associated in one cluster present homogeneous stable isotopic ratios over space.

Results of simulations are given by proportions of sources expressed as percentages. Although in order to compare, in each hominid group and in each simulation, the contribution of each source to the mixture, Chi-squared test with a Bonferroni correction was used. To use the Chi-squared test, a standardisation of results has been done based on a calculation of a resource unit related to the quantity of protein intake.

## 3. Results

### 3.1. Isotopic Variability Analyses

A descriptive statistical analysis of the complete isotopic dataset was performed to detect whether isotopic differences existed (i) between the archaeological sites of the considered geographic region, (ii) between the archaeological sites of the considered environments as well as (iii) between chronological subdivisions of MOIS3 and MOIS2. Kruskal-Wallis analyses were implemented on the whole dataset, on the South-western dataset, and on the cold environment dataset to test if chronological groups are efficient and if all archaeological sites associated with a chronological group (MOIS3 Neanderthal, MOIS3 modern humans, or MOIS2 modern humans) could be pooled together in terms of carbon and nitrogen isotopic ratios. *P* values lower than 0.01 indicate statistically significant differences suggesting an isotopic heterogeneity within groups.

1. Regarding the analysis of the complete dataset (Table SI4), we often observed a particular dietary/isotopic behaviour for the reindeer which seems attributed to local environment. This is why we decided to perform two sets of simulations, a set including the reindeer and a set excluding from the reindeer. Concerning MOIS2 whole dataset, bovids and horse show isotopic variability across archaeological sites. We identified and excluded the divergent archaeological sites for bovids (Gough's cave and Kendrick's cave for bovids). Since divergent archaeological sites were not identifiable for horse, in order to avoid any biases, several sets of simulations were conducted with different *δ* ^15^N values for herbivorous sources (*δ* ^15^N ± 1SD; Figure 2).
2. For the South-western area (Table SI5), reindeer isotopic variability seems attributed to local environment whereas bovids' seems to be heterogeneous during MOIS2. To avoid any biases, divergent sites have been excluded from the simulations (Pont d'Ambon, [31]). As mentioned above, additional simulations have also been performed with and without reindeer.
3. In cold environments (Table SI6), reindeer isotopic variability seems also attributed to local environment. Similarly, simulations with and without consideration of the reindeer isotopic variability within the herbivorous group were conducted.

To summarise, based on the results of Kruskal-Wallis analyses, simulations were performed according to each cluster and each chronological group under several conditions: (1) presence of the three faunal types in the herbivorous source (bovid, reindeer, horse), (2) presence of bovids and horse, in the herbivorous source, to take into account the isotopic variability of reindeer across archaeological sites), (3) presence of bovids and reindeer in the herbivorous source, to take into account that horses are nonruminant species [81]. For the environmental cluster only, (4) a fourth simulation was performed considering only the reindeer in the herbivorous source since it was probably the main species consumed under these cold climates [82].

In addition, two sets of simulations were performed to guaranty the most reliable application of isotopic biochemistry in the reconstruction of past diet: (1) one considering the totality of the hominids for each chronological group and (2) a second considering only the hominids associated with fauna. In total, 19 simulations have been performed on the whole dataset, 28 on the South-western dataset, and 16 on the cold environment dataset (Table 4).

### 3.2. Simulation Results

Simulations were implemented using three food sources selected according to isotopic dataset relative to each cluster and each chronological group. In all simulations, source 1 shows fixed values characterised by high *δ* ^15^N values and low *δ* ^13^C values. Source 2 and source 3 isotopic signatures were determined according to isotopic dataset relative to each cluster and each chronological group. In all simulations, source 2 shows intermediate *δ* ^15^N values and high *δ* ^13^C values whereas source 3 exhibits low *δ* ^13^C and *δ* ^15^N values. The result of the simulations is driven by the relative position of the mixture compared to the three sources.

Regarding the MOIS3 Neanderthals' dataset (Table 5), in all clusters and under all conditions, the results of the different simulations show the same patterns. The contribution of source 1 is the highest (between 48% and 67% of the mixing food), suggesting a consumption of food with high *δ* ^15^N values and low *δ* ^13^C values. The contribution of source 3 (between 28% and 44%) is lower than source 1 but higher than the contribution of source 2 (between 1% and 19%), which confirms the greater consumption of food with low *δ* ^13^C signatures. More precisely, simulations without considering the reindeer isotopic values tend to increase the contribution of source 2, whereas simulations without considering the horse isotopic values tend to decrease the contribution of source 2. In other words, the more the isotopic value of the herbivorous source is enriched in ^13^C, the fewer source 2 contributes to the mixture. In the South-western area, even if the contribution of source 2 slightly decreases when simulations are based on average isotopic values of different sources, the differences between the two sets of simulations (regarding a large variability versus an average variability) are not significant.

Concerning the MOIS3 modern humans' dataset (Table 6), the results of simulations within the South-western area exhibit different dietary patterns relatively to the other two clusters (global and environmental). For the three clusters, the contribution of source 1 is the highest (between 58% and 72%). In the South-western area, the contribution of source 2 is higher than the contribution of source 3 (resp., between 15% and 40% and between 1% and 13%). It is the opposite for the global and environmental clusters, the contribution of source 3 is higher than the contribution of source 2 (resp., between 26% and 32% and between 2% and 15%). These results would suggest a lesser consumption of ^13^C enriched food. Regardless of the faunal types considered in the herbivorous source, the results globally remain the same for the cold environments and the complete datasets. Nevertheless, when the reindeers are absent, the contribution of source 3 is lower than the contribution of source 2. It is the opposite when horses are not considered in the simulations. 

In the South-western area, similar dietary patterns are observed when the reindeers are not considered; the contribution of source 3 clearly decreases whereas the contribution of source 2 clearly increases. It is also the opposite when horses are not considered in simulations. As suggested for MOIS3 Neanderthals, we can suppose that the more the isotopic values of herbivorous source are enriched in ^13^C, the fewer source 2 contributes to the mixture. The divergent results observed for the South-western area regarding the relative contribution of source 2 and 3 could be explained by a bias induced by the isotopic value of the mixture representing only by one available data in the South-western area for this period. Differences between the two sets of simulations for the South-western area are low. The contribution of source 3 slightly decreases when simulations are based on average isotopic values of the three sources whereas the contribution of source 2 increases.

Concerning the MOIS2 modern humans dataset (Table 7), independently of clusters and contrary to the MOIS3 Neanderthals and modern humans, dietary patterns show a slight predominance of source 2 (between 20% and 82%) compared with the other two sources (between 0 and 40% for source 3 and between 30% and 51% for source 1). Some differences have been observed according to faunal types included in the herbivorous source. Overall, the contribution of source 3 slightly decreases when the reindeer are excluded and increases when the horse are excluded. These different dietary patterns according to the inclusion or exclusion of reindeer and horse in herbivorous source are consistent with the observations made for MOIS3 Neanderthals and MOIS3 modern humans. Regarding the two sets of simulations performed for the South-western area, the contribution of source 3 decreases in conjunction with an increase of source 2 when average isotopic values are considered for sources. An exception is observed when horses are not included in the herbivorous source. The contribution of source 2 also increases when the herbivorous source exhibits high *δ* ^13^C values.

Chi-squared tests were conducted according to the three chronological groups. The results of this statistical analysis emphasize previous observations (Table 8). With the Bonferroni correction and two degrees of freedom, the *P* values are considered significant below 0.017. Dietary patterns of MOIS2 modern humans appear statistically different than the other two chronological groups. Dietary patterns of MOIS3 modern humans and MOIS3 Neanderthals do not exhibit significant differences for global and environmental clusters. For geographical cluster, as observed on raw results, simulations show significant differences in dietary patterns between MOIS3 modern humans and MOIS3 Neanderthals. As mentioned before, this could be explained by the unique nitrogen isotopic value available for MOIS3 South-western modern humans.

## 4. Discussion and Conclusions

Our research on past human diet during the transition from MOIS3 to MOIS2 is based on the modelling of isotopic signatures of a mixture over time and under several clusters (global, geographic, or environmental clusters). Our aim has been to test the hypothesis that resource competition, analysed through isotopic modelling, may have existed between Neanderthals and contemporaneous modern humans. Isotopic analyses, which are generally used for studying local environments, would also seem to be applicable to a population approach. Indeed, the results of our modelling illustrate that, whatever cluster is considered, the dietary behaviour of each chronological group shows similar dietary patterns. In order to compensate for the lack of reliability of certain sets of data, modelling seems to be a relevant approach. 

In agreement with the hypotheses underlying our models, we were able to compare MOIS3 modern humans with the two other hominid groups (MOIS3 Neanderthals & MOIS2 modern humans), even if hominid isotopic values for MOIS3 modern humans considered in simulations were not associated with the faunal isotopic values. Indeed, results of our modelling show that whatever the conditions considered (hominids with and without associated fauna), the dietary behaviour of each chronological group shows similar patterns. 

Concerning the diachronic analysis of past dietary patterns, our study demonstrates the absence of significant differences between Neanderthal diet and that of contemporaneous modern human and highlights the dietary difference among MOIS2 modern human. These conclusions confirm that resource competition may have occurred during MOIS3 between the two hominid populations living in Europe. Some authors have already suggested competition between the two populations by observing a correspondence between the contraction of the Neanderthal ecological niche and the expansion of the ecological niche of modern humans [9]. However, radiocarbon dates do not provide any cases of geological interstratification (shown by Mousterian, Aurignacian, and transitional assemblages), which would support the contemporaneity of Neanderthals and modern humans [3]. It has been argued that much of Europe was almost empty when the modern human expansion occurred [7, 83]. Thus, given the radiocarbon dates, little contact would occur between the two populations in Europe, except in the Southwest of France and in the North of Spain, where encounters may have been more frequent [83]. Therefore, resource competition would have happened only in these areas of contact. 

The divergence of MOIS2 modern human behaviour in relation to MOIS3 populations (Neanderthals and modern humans) may be explained through use by the former population of alternative food sources, such as, for example, small fauna. Our results are consistent with the observation of certain prehistorians, who suggest a relative continuity in behavior between Neanderthals and contemporaneous modern humans and a behavioural modification between Aurignacian and Gravettian modern humans [84, 85]. Most publications on modern human dietary spectra have underlined that this population had a more diverse diet than Neanderthals, consuming, beside ungulates, small game prey like fish or small mammals [19, 20, 24, 86–88]. Due to the lack of data, stable isotopic signatures of the small prey were not considered in our simulations, and it is therefore difficult to reach any conclusions concerning potential modifications in the consumption of this type of prey. Nevertheless, if small prey have similar isotopic signatures to those of reindeer, bovids, and horse used here, our results would explain the difference in patterns observed for MOIS2 modern humans (with a higher contribution of source 2) in comparison to MOIS3 Neanderthals and MOIS3 modern humans. On the contrary, the predominance of source 1 in MOIS3 Neanderthals and MOIS3 modern humans could indicate a consumption of food with high *δ* ^15^N values and low *δ* ^13^C values. Source 1 considered in our simulations was derived from fish isotopic values, although other species with similar isotopic value, such as mammoths [19], may have been taken into account and used instead of fish.

Furthermore, zooarchaeological and genetic studies indicate a decline in the quantity of big game and notably ungulates, beginning at about 50,000 years BP [89–91]. On the basis of our results, Neanderthals did not change their diet during MOIS3, as Richards and Trinkaus have already suggested in 2009 [92]. Prior to the early modern human expansion, it is possible that Neanderthals, which had a small population size [93–98], were not affected by a reduction of the population size of large mammals. By contrast, during the period of coexistence of Neanderthals and modern humans, the MOIS3, we can suppose that faunal contraction, associated with resource competition, might have had more serious consequences. Indeed, Neanderthals were probably close to their carrying capacity due to the decrease of the ungulate populations [99], as is suggested by certain stress markers (dental hypoplasia, etc.) [100, 101]. In such conditions, the arrival of species evolving in a same ecological niche might have led to strong competition and perhaps contributed to the demise of Neanderthals, although competition alone cannot account for Neanderthal extinction. Some authors suggest that early modern humans would have possessed more varied technical abilities [10, 102–104], were able to adjust their hunting toward a more varied prey [5], and would have required less food and fewer foraging returns [105, 106]. All of these factors may have favoured the modern humans' survival [10].

In conclusion, our study has adopted the hypothesis that all the hominids we have considered consumed the same kind of resources. Provided that this hypothesis does not involve a major bias, our methodology, based upon modelling, has permitted us to address the question concerning resource competition between Neanderthals and modern humans, to which zooarchaeological approaches, in view of the complexity of faunal assemblages, could not supply a clear answer [5]. Thanks to a substantial isotopic database, this study confirms the occurrence of resource competition between Neanderthals and contemporaneous modern humans living in the same area. It is however not possible to reach a conclusion concerning the role of competition in Neanderthals' demise. Furthermore, this study underlines the dietary changes that occurred during MOIS2, as proposed by the study of lithic industry and archaeological data of faunal assemblage. It also demonstrates that modelling approaches and dietary assessment are useful for investigating ecological interaction among both present and past populations. In order to answer this last question, we are engaged in a study in progress, which uses complex mathematical models to represent, as plausibly as possible, the trophic web of Neanderthals and the interaction between prey, predators, and hominid groups (Neanderthals and early modern humans).

## Figures and Tables

**Table 1:** Palaeolithic isotopic data available for modelling.

Sample	Number of data	References
Reindeer	201	[20, 31–37]
Horse	230	[17–20, 31, 33–44]
Bovid	88	[18–21, 31, 33–37, 39, 41–43, 45–47]
Neanderthal	19	[16, 18–21, 31, 35–37, 42, 47–51]
Modern human	61	[16, 17, 22, 31, 33, 34, 46, 52–61]

**Table 2:** Palaeolithic isotopic data available for modelling distributed according to geographical cluster and environmental cluster (each subcluster contains at least one hominid record).

Clusters	Geography	*N*	Environment	*N*
Sub-clusters	North-west Europe	135	Tundra-steppe	50
	South-west Europe	447	Open boreal woodland	99
	Central	17	Tundra/boreal woodland	84
			Steppe	12
			Cold steppe	151
			Wooded steppe	47
			Temperate forest	24
			Warm (wooded) steppe	36
			Undefined	96
Total		*599*		*599*

**Table 3:** Isotopic data available for each chronological group considering (1) geographical cluster and (2) environmental cluster. The three conditions retained for isotopic simulations are marked by grey cells.

Chronological groups	MOIS3 Neanderthals		MOIS3 modern humans		MOIS2 modern humans	
	Hominids	Faunas	Hominids	Faunas	Hominids	Faunas
Geographical regions						
North-west Europe	×	×			×	×
South-west Europe	×	×	×	×	×	×
Central	×	×	×		×	×
Environments						
Tundra-steppe	×	×			×	×
Open boreal woodland	×	×			×	×
Tundra/boreal woodland	×	×	×	×	×	×
Steppe					×	×
Cold steppe					×	×
Wooded steppe	×	×			×	×
Temperate forest					×	×
Warm (wooded) steppe					×	×
Totality of the dataset	×	×	×	×	×	×

**Table 4:** Characterization of the simulations performed with IsoSource according to the different geographical groups and the three clusters considered.

		Simulations											
Conditions tested		Whole fauna/whole hominids	Whole fauna/associated hominids	Fauna without reindeer/whole hominids	Fauna without reindeer/associated hominids	Fauna without horse/whole hominids	Fauna without horse/associated hominids	Reindeer/whole hominids	Reindeer/associated hominids	Whole fauna (*δ* ^15^N − 1SD)/whole hominids	Whole fauna (*δ* ^15^N − 1SD)/associated hominids	Whole fauna (*δ* ^15^N + 1SD)/whole hominids	Whole fauna (*δ* ^15^N + 1SD)/associated hominids

MOIS3 Neanderthals	Southern Europe area	X	X	X	X	X	X						
	Cold environment	X	X	X	X	X	X	X	X				
	Whole dataset	X	X	X	X	X	X						

MOIS3 modern humans	Southern Europe area	X		X		X							
	Cold environment	X		X		X		X					
	Whole dataset	X		X		X							

MOIS2 modern humans	Southern Europe area	X	X	X	X	X	X						
	Cold environment	X	X			X	X	X	X				
	Whole dataset	X	X	X	X	X	X			X	X	X	X

**Table 5:** Results of simulations by IsoSource for MOIS3 Neanderthals chronological group and under the three clusters; circular diagram represents the different source proportions to the mixture.

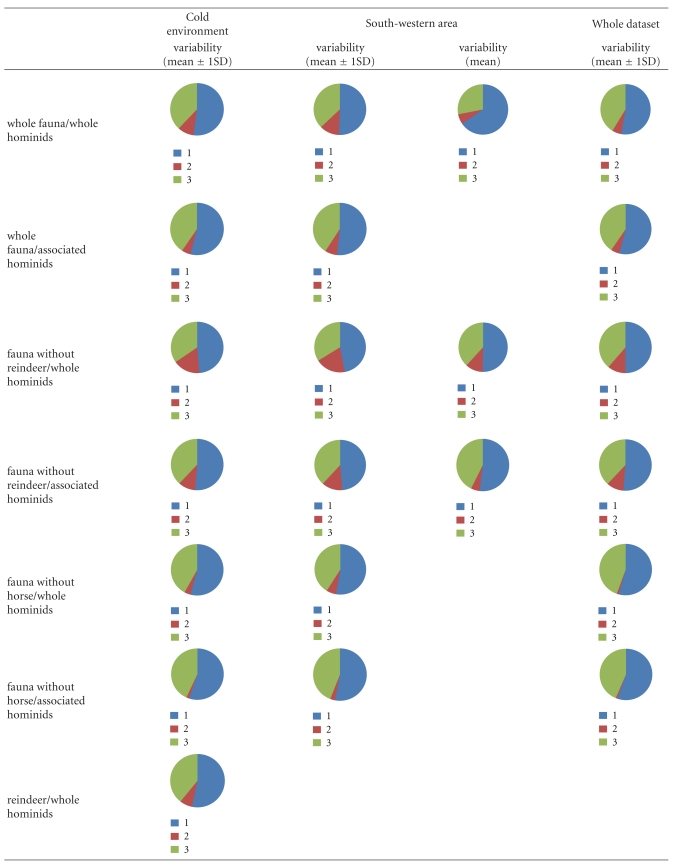 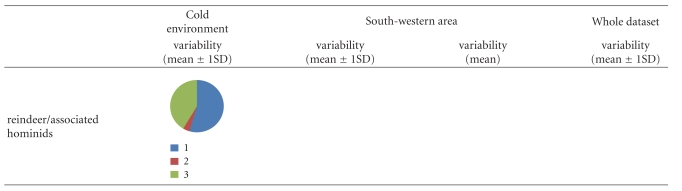

**Table 6:** Results of simulations by IsoSource for MOIS3 modern humans chronological group and under the three clusters; circular diagram represents the different source proportions to the mixture.

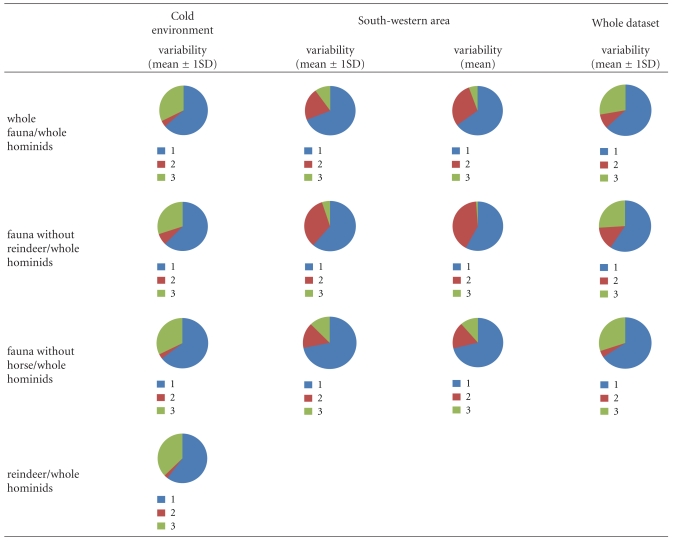

**Table 7:** Results of simulations by IsoSource for MOIS2 modern humans chronological group and under the three clusters; circular diagram represents the different source proportions to the mixture.

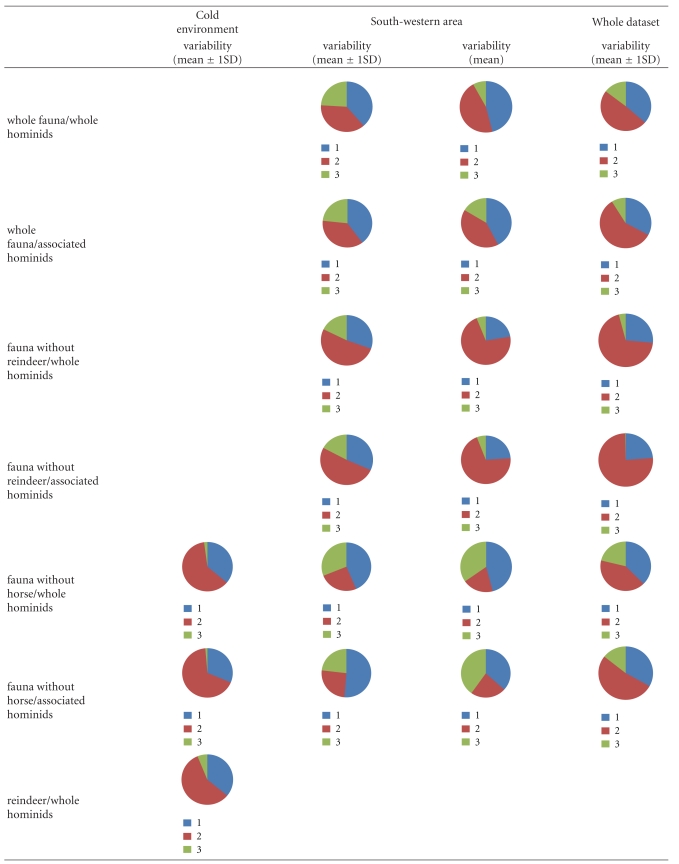 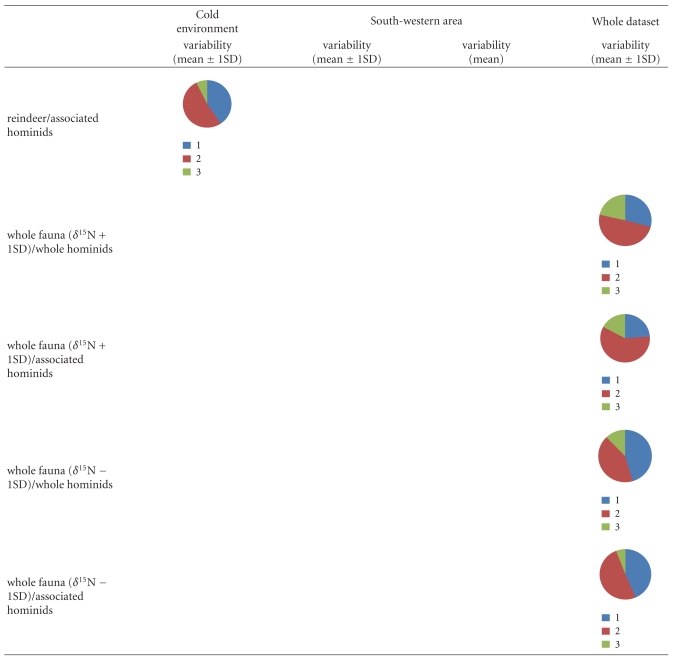

**Table 8:** Chi-squared results, significant values of *P* (<0.017) are in italic.

				N3-MH3	N3-MH2	MH3-MH2
South-western area	Extended variability	Whole fauna	Whole hominids	0.000038	0.000000	0.000000
		Fauna without reindeer	Whole hominids	0.000002	0.000000	0.000000
		Fauna without horse	Whole hominids	0.000007	0.000000	0.000003
	Average variability	Whole fauna	Whole hominids	0.000000	0.000000	0.000382
		Fauna without reindeer	Whole hominids	0.000000	0.000000	0.000000
		Fauna without horse	Whole hominids	NA	0.000000	0.000059

Cold environment	Extended variability	Whole fauna	Whole hominids	*0.015359*	0.000000	0.000000
		Fauna without reindeer	Whole hominids	0.002858	NA	NA
		Fauna without horse	Whole hominids	NA	0.000000	NA
		Reindeer	Whole hominids	*0.031744*	0.000000	0.000000

Whole dataset	Extended variability	Whole fauna	Whole hominids	*0.102942*	0.000000	0.000000
		Fauna without reindeer	Whole hominids	*0.143751*	0.000000	0.000000
		Fauna without horse	Whole hominids	*0.026514*	0.000000	0.000000
